# Fitness Costs in Diamondback Moth *Plutella xylostella* (L.) (Lepidoptera: Plutellidae) Resistant to Lufenuron, A Chitin-Synthesis Inhibitor Insecticide

**DOI:** 10.3390/insects15110856

**Published:** 2024-11-02

**Authors:** Natalia C. Bermúdez, Nataly de la Pava, Deividy V. Nascimento, Lilian M. S. Ribeiro, Herbert A. A. Siqueira, Jorge B. Torres

**Affiliations:** 1 Departamento de Agronomia-Entomologia, Universidade Federal Rural de Pernambuco, Rua Dom Manoel de Medeiros s/n, Dois irmãos, 52171-900 Recife, PE, Brazil; nataliabermudezbuitrago@gmail.com (N.C.B.); deividysx@gmail.com (D.V.N.); lilian_biology@yahoo.com.br (L.M.S.R.); herbert.siqueira@ufrpe.br (H.A.A.S.); 2 Facultad de Ingeniería Programa de Ingeniería Agronómica, Universidad del Magdalena, Calle 29H3 No 22-01, Santa Marta 470004, Colombia; natalydlp@gmail.com

**Keywords:** diamondback moth, life table, benzoylureas, fitness costs, demography

## Abstract

The diamondback moth (DBM), *Plutella xylostella,* is the main pest of Brassicas crops worldwide, and its recorded resistance to 101 active ingredients indicates it is difficult to control. The purpose of this study was to investigate the hypothesis that *P. xylostella* has fitness costs associated with its resistance to lufenuron, a chitin-synthesis inhibitor insecticide. Thus, concentration–mortality bioassays were performed for susceptible (REC-S), resistant (BZR-R) populations, their progenies F1 and F1′, and one established population without selection pressure (BZR-Rns) after four generations. A fertility life table was used to assess the biological performance of the REC-S and BZR-R. BZR-Rns of *P. xylostella*. The larval stage, longevity, and survival differed between populations. The reproductive rate (R_0_) was significantly lower in the F1 (♀R × ♂S) (28.19) and F1′ (♀S × ♂R) (34.06) progenies compared with their parents, but not with the relaxed BZR-Rns (39.39). The mean generation time (T), intrinsic rate of population growth (r_m_), and doubling time (DT) differed between REC-S and progenies, with fitness of 0.52 and 0.64 for F1 and F1′, respectively. Overall, the results suggest that the resistance of *P. xylostella* to lufenuron is stable and that low fitness costs appear to be associated with resistance to lufenuron, although heterozygotes showed lower fitness than their parents. Strategies such as preserving refuge areas, rotation of modes of action, etc., are essential for resistance management and prolonging the efficacy of control agents; this highlights the importance of integrated insecticide resistance management.

## Simple Summary

Resistance to insecticides is one of the main factors that makes it difficult to control the diamondback moth, *Plutella xylostella*, on Brassica crops. Recurrent control failures lead to more frequent insecticide applications, use of higher doses, and decreased yield. This results in environmental pollution, increased population resistance, and economic loss. Studies that elucidate the characteristics of resistance are necessary to improve pest control strategies and overcome this problem. Resistance, in turn, carries a fitness cost, which impairs the biological characteristics, or fitness, of the resistant population compared to a susceptible population in environments without insecticides. Thus, the present study evaluated the fitness cost of resistance to lufenuron in a *P. xylostella* population, as well as the stability of this resistance in the resistant population without selection pressure for four generations. As a result, we observed that the resistant population demonstrated greater resistance than the susceptible population of *P. xylostella*, as well as a low fitness cost which was associated with lufenuron resistance. This information supports the adoption of strategies such as preserving refuge areas near crops for susceptible insects and natural enemies, helping to dilute resistance in the field, as well as other strategies.

## 1. Introduction

The annual cost of managing the diamondback moth (DBM), *Plutella xylostella* (L.) (Lepidoptera: Plutellidae), is estimated to be USD 4–5 billion per year [1], with insecticide application accounting for a significant portion of this cost. This cost, along with crop losses, damage to all cultivated Brassicas, and distribution, elevates the DBM to the status of the world’s leading Brassica pest. Insecticide resistance is considered the main challenge when it comes to controlling *P. xylostella*. This misleads growers into spraying insecticides more frequently to achieve effective control and reduce crop losses. Resistance records are common in the field due to the pest’s high selection pressure and genetic plasticity, which make *P. xylostella* resistant to most insecticides used for its control (https://www.pesticideresistance.org/ (accessed on 17 July 2024)). For instance, in 2024, researchers reported that *P. xylostella* demonstrated resistance to 101 active ingredients from various insecticide groups, such as organochlorines, organophosphates, carbamates, pyrethroids, nereistoxin analogues, *Bacillus thuringiensis* (Bt), ivermectin, spinosyns, phenylpyrazoles, oxadiazines, bisacylhydrazines, diamides, and benzoylureas [2].

Lufenuron is a selective insecticide that is primarily recommended for use against insect larvae, including diverse species of Lepidoptera, Coleoptera, Diptera, and, to a lesser extent, thrips, hemipterans, and mites. Lufenuron’s selectivity is an important characteristic for integrated pest control. As an insect growth disruptor (IGD), it inhibits chitin synthesis during the moulting process [3,4]. It directly interacts with the chitin synthase 1 (CHS1) protein, inhibiting the proper polymerization of chitin [5]. Selection pressures exerted by IGDs have led to resistance development in certain populations of *P. xylostella*, either through metabolic detoxification via glutathione S-transferase [6] or through a mutation (I1042M/F) at position 1042 in the terminal region of the protein, which confers resistance to benzoylureas [3].

In Brazil, the first report of resistance to lufenuron in a *P. xylostella* population was made in Pernambuco as a result of the indiscriminate use of this insecticide in that region [7]. A late study on a population from this location had a resistance ratio (RR) of 11,283-fold compared to a reference population for susceptibility. This resistance was linked to an autosomal, incompletely recessive, and single-factorial trait [8]. Field-evolved resistance to lufenuron has also been reported in other Lepidoptera species such as *Spodoptera exigua* (Hubner) [9], *Spodoptera litura* (Fabr.) [10], *Spodoptera frugiperda* (J.E. Smith) [11,12], *Chrysodeixis includes* (Walker) [13], as well as in species from other orders such as *Musca domestica* (L.) (Diptera: Muscidae) [14] and *Phenacocccus solenopsis* (Tinsley) (Hemiptera: Pseudococcidae) [15]. Despite this, there have only been a few other reports of IGD resistance (https://www.pesticideresistance.org/ (accessed on 24 March 2024)), underscoring the importance of our findings and their potential for improving our understanding of the evolution of lufenuron resistance in parallel with other IGDs.

It is well known that the development of insecticide resistance generally results in fitness costs in the resistant population in the absence of insecticide exposure compared to the susceptible population [16]. There are various ways to express this phenomenon. For instance, reduced fecundity and fertility, prolonged development time, and reduced life expectancy can reflect the fitness cost of an insecticide-resistant population [17,18]. Furthermore, fitness costs are also related to the rapid decline of the resistance rate in a population [19]. Previous studies conducted directly on *P. xylostella* have shown that fitness costs, such as delayed developmental time, decreased weight in different instars, decreased oviposition period, fecundity, and fertility, are a direct consequence of insecticide resistance [20,21,22,23]. In addition, reduced fitness can also occur in progenies resulting from crosses between susceptible and resistant species [20,24]. When fitness costs are found in resistant populations, management tactics such as multiple-insecticide attacks and mode of action rotation are suggested, as they result in more effective responses.

Understanding the fitness cost within a resistant population helps us to develop effective management tactics that exploit the possible weaknesses of the resistant population and, consequently, to address resistance in the field and extend the shelf life of a pesticide in the market. Thus, in order to clarify the effects of lufenuron resistance in *P. xylostella*, this study aimed to evaluate the existence of fitness costs in a lufenuron-resistant population compared to a reference for susceptibility, as well as their progenies.

## 2. Materials and Methods

### 2.1. Insects

This study initially used two populations of DBM, one susceptible and the other resistant. The susceptible population (hereafter named as REC-S) was collected from organic cultivated Brassicas (8°15′19″ S, 35°29′46″ W) located in Chã Grande County, Pernambuco State, and was reared in a laboratory without any contact with insecticides. The resistant population (hereafter named as BZR-R) originated from larvae collected from a cabbage crop (8°14′33″ S, 35°47′7″ W) located in Bezerros County, Pernambuco State, Brazil. To keep the BZR-R population under the selection pressure for 34 generations, second-instar larvae were fed cabbage leaves that had been dipped into 1000 mg of lufenuron (Match™ 50EC, Syngenta S.A., São Paulo, SP, Brazil) L^−1^ dilution. Collard greens (*B. oleracea* var. *acephala*) leaf discs of 9 cm in diameter were submerged for ten seconds in a concentration of 1000 mg lufenuron L^−1^ combined with Triton™ X-100 (Sigma-Aldrich, St. Louis, MO, USA) (0.01%) and allowed to dry at laboratory temperature on paper towels for ≈ 2 h. The concentration used was based on preliminary concentration-response tests, where the median lethal concentration (LC_50_) was estimated, and used to apply selection pressure to each generation. The REC-S larvae were reared on collard greens leaves without any insecticide treatment. In order to evaluate the fitness of the progenies and after the phenotypic differentiation between the populations mentioned above, reciprocal crosses were made between virgin males (n = 50) and females (n = 50) of the REC-S and BZR-R populations to obtain the progenies F1 (♀BZR-R × ♂REC-S) and F1′ (♀REC-S × ♂BZR-R). Sex differentiation was conducted during the final larval stage, as this is when the male gonads are visible. We also assessed the resistant population for four successive generations without selection pressure (hereafter named as BZR-Rns), following the division of the resistant population into two. The larvae were fed with collard greens leaves without insecticide. Adults were offered a 10% honey solution for food and collard greens leaves as a substrate for oviposition [25].

### 2.2. Susceptibility Bioassays

Concentration–mortality bioassays were conducted for each previously described DBM population using the IRAC bioassay method #018 (https://irac-online.org/test-methods/ (accessed on 28 March 2018 )) to assess the LC_50_. Collard greens leaf discs (5 cm in diameter) were washed with 5% sodium hypochlorite, then rinsed with tap water, and then washed again with distilled water. Subsequently, the leaf discs were submerged for ten seconds in difference concentrations of lufenuron combined with Triton™ X-100 (0.01%) and allowed to dry at laboratory temperature on paper towels. The control group included collard greens leaf discs treated only with distilled water and Triton™ X-100 (0.01%). Upon drying, the treated leaf discs were placed in Petri dishes (60 × 15 mm) laid on filter paper (5 cm) saturated with 100 µL of distilled water. Ten second-instar DBM larvae were placed on each plate (replication) and a total of three replications were used for each concentration. The samples were maintained in a climatic chamber (Marconi S.A. model MA 403, Piracicaba, SP, Brazil) regulated at 25 ± 1 °C, 65 ± 5% relative humidity (RH), and a photoperiod of 12 h. We assessed mortality 96 h post-exposure, censoring only the larvae that were either dead or alive, resulting in an observation of 27 to 30 larvae per treatment. Table 1 displays the total count of larvae recorded for each bioassay. The assessment criterion relied on the larvae’s capacity to move a distance equivalent to their body length in response to prodding with a soft bristle brush (#3/0).

### 2.3. Biological Characteristics of Immatures and Adults of DBM-Resistant, -Susceptible, and Progeny Populations

The fitness comparison experiments were conducted under controlled conditions in a climatic chamber regulated at 25 ± 1 °C, 65 ± 5% RH, and a 12 h photoperiod. A 5 cm diameter Petri dish containing a disc of collard greens leaf of the same diameter placed over filter paper saturated with 100 µL of distilled water was used to rear *P. xylostella* larvae at rate of 5 larvae per dish. The developmental time was assessed daily when collard greens leaves were replaced with fresh leaves. Each population included a total of 125 larvae. Upon reaching the fourth instar, the larvae were sexed and kept in the same Petri dish until they reached the pupal stage.

The pupae were collected and placed individually in test tubes sealed with cotton to allow for gas exchange; subsequently, the pupae were weighed 24 h post-collection. Eleven pairs of males and females from each population were assembled and each couple reared within a cage to assess egg production. The individual adult rearing cages were made using 300 mL plastic cups, as outlined in the methods of Barros and Vendramim [25]. Each cage had a daily provision of a 5 cm diameter leaf disc cut from collard greens leaf offered as a substrate for oviposition until the last moth died. The number of eggs laid was tallied every 24 h when the leaf discs were replaced with fresh leaf discs. The leaf discs containing the eggs were incubated and the number of larvae hatched was recorded two days later. A sample of the first instar larvae was reared as previously described to assess their performance. Pupae were collected and individually reared in test tubes sealed with cotton to allow for gas exchange. Upon the emergence of the adults, we verified their sex and documented data to compute the sex ratio.

### 2.4. Fertility Life Table

The population growth parameters, the net reproductive rate (R_0_), the mean generation time (T), the intrinsic rate of population growth (r_m_), and the population doubling time (DT) were estimated. The parameter estimations used data on developmental times and viability for the whole immature stage, sex ratio, female daily fecundity, and adult survival. The calculations used to build the fertility life table followed the formulae described by Krebs [27] and used by Maia et al. [28] to write the LIFETEST procedure in SAS. Then, the life table parameters were calculated by adapting the procedure described by Maia et al. [28] using the estimator Jackknife method performed by SAS software 9.4 (SAS Institute, Cary, NC, USA). The determined parameters were compared pairwise using the overlap rule of the confidence interval (CI) at 95% probability [29]. The relative fitness of the resistant strain was calculated as Rf = R_0_ of the test population/R_0_ of the reference population for susceptibility. Rf > 1 indicates that the resistant population has positive fitness, whereas Rf < 1 indicates that the resistant population has reduced fitness [30].

### 2.5. Data Analysis

The mortality data were submitted to Probit’s analysis [31] after being corrected for the natural mortality observed in the control group [32] using POLO-Plus software (LeOra Software Inc., Petaluma, CA, USA). The RR was determined using the LC_50_ and was considered significant when the 95% fiducial limit (FL) did not include a value of 1 [26]. To ascertain the similarities or significant differences between the populations of *P. xylostella*, data that did not assume normality (larval period, pupal period, and pupal weight) were subjected to the Kruskal–Wallis’ test, followed by the Wilcoxon–Mann–Whitney test at 5% probability. The ANOVA (PROC D.F.M.) was used to analyze data that fit normality, including longevity, period of oviposition, fecundity, and fertility, followed by Tukey’s test (HSD) to separate the mean values. To test the difference in the pupae viability and sex ratio between populations, the data were submitted to PROC FREQ of SAS. The Kaplan–Meier method (PROC LIFETEST) was used to calculate the adult survival curves, and the Log-Rank test (α = 0.05) was applied to compare them. We analyzed all data using the SAS software (SAS Institute).

## 3. Results

### 3.1. Susceptibility Bioassays

The mortality response of DBM larvae to lufenuron concentrations fitted the Probit model (*p* > 0.05). The susceptible population REC-S had an LC_50_ value of 0.71 mg lufenuron L^−1^, contrasting with the resistant population BZR-R, which had an LC_50_ value of 870.5 mg lufenuron L^−1^. The LC_50_ values of the F1′ and F1 progenies were 2.12 and 3.80 mg lufenuron L^−1^, respectively. The bioassay with the BZR-Rns population yielded a LC_50_ of 479.37 mg lufenuron L^−1^ (Table 1). The calculated RR between BZR-R and REC-S populations was 1224.26 times, whereas the BZR-Rns population exhibited an RR of 674.19 times. The RR values for the F1 and F1′ progenies were 5.35 and 2.98 times, respectively (Table 1).

### 3.2. Biological Characteristics of Immatures and Adults of DBM-Resistant, -Susceptible, and Progeny Populations

The developmental time of larvae varied across DBM populations (χ^2^ = 156.47; df = 4; *p* < 0.0001). The larval stage of the REC-S population lasted less time than the larval stages of the BZR-R (χ^2^ = 30.70; df = 1; *p* < 0.0001) and BZR-Rns populations (χ^2^ = 114.11; df = 1; *p* < 0.0001), F1 progeny (χ^2^ = 70.59; df = 1; *p* < 0.0001) and F1′ progeny (χ^2^ = 86.19; df = 1; *p* < 0.0001). Likewise, the developmental time of BZR-R larvae was shorter than that of BZR-Rns (χ^2^ = 30.58; df = 1; *p* <0.0001). The developmental times of larvae from progenies F1 and F1′ did not differ from each other (χ^2^ = 21.74; df = 1; *p* = 0.641). However, these larvae exhibited a longer developmental time compared to BZR-R (χ^2^ = 38.77; df = 2; *p* < 0.0001) and BZR-Rns populations (χ^2^ = 9.47; df = 2; *p* = 0.0087) (Table 2).

The weight of 48 h old pupae did not differ among populations (*p* > 0.05), except that the pupae from BZR-R that were smaller than pupae of REC-S (χ^2^ = 10.53; df = 1; *p =* 0.03). Furthermore, the rate of adult emergence was between 78.6 and 93.3% and did not differ among populations (χ^2^ = 14.74; df = 4; *p >* 0.05).

Adult female longevity was similar among populations (F = 1.87; df = 4; *p* = 0.13), whereas male longevity varied (F = 4.76; df = 4; *p* < 0.005). Males from the resistant population BZR-R survived longer than adults from F1′, F1, and BZR-Rns; however, the same was not true of males from REC-S (Table 2).

The time needed to complete the pupal stage differed across populations (χ^2^ = 14.74; df = 4, *p* = 0.005). Nevertheless, when the degree of freedom was reduced to 1, pairwise comparisons revealed no significant difference between pupal stages of REC-S and BZR-Rns (χ^2^ = 1.85; df = 1, p = 0.173) or their progenies F1 (χ^2^ = 2.83; df = 1; *p* = 0.092) and F1′ (χ^2^ = 0.0075; df = 1; *p* = 0.093), except for a close difference with BZR-R (χ^2^ = 3.41; df = 1; *p* = 0.064).

The proportion of females that emerged was between 48.6 and 58.7%, and it was similar across populations (*p* > 0.05). Likewise, the oviposition period was similar among populations (*p* > 0.05) between 7.2 and 9.3 days. Nevertheless, the number of eggs produced (fecundity) differed among populations (F = 4.27; df = 4; *p* = 0.005). Females from the progeny F1 produced more eggs than those from the progeny F1′ and BZR-Rns, but their egg production was similar to that of the parental females from REC-S and BZR-R. In addition, the egg hatching rate (fertility) was different among populations (F = 2.89, df = 4; *p* < 0.05). Eggs laid by females in the progeny F1 exhibited higher viability than eggs laid by females in the population BZR-Rns, but there was no difference among egg viability for other populations (Table 2).

**Figure 1:**
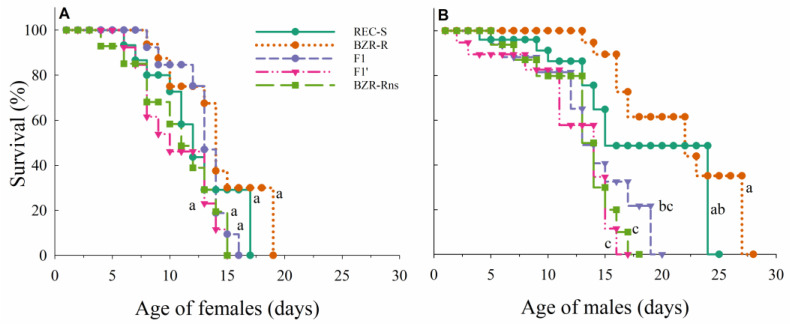
Survival (%) of adult females (**A**) and males (**B**) of *Plutella xylostella* from the populations that were susceptible (REC-S) and resistant (BZR-R) to lufenuron, and for the reciprocal crosses F1 and F1′ and resistant population without selection pressure (BZR-Rns). Different letters indicate a significant difference in survival according to the Log-Rank test (α = 0.05).

The females from different populations exhibited comparable survival patterns (χ^2^ = 4.42; df = 4; *p* = 0.35) (Figure 1A). Males’ survival patterns, however, varied among populations (χ^2^ = 27.67; df = 4; p < 0.0001) (Figure 1B). Males from the resistant population BZR-R lived longer than males in the progenies F1 and F1′, as well as the population BZR-Rns. Males from the REC-S population also lived longer than males from progeny F1′ and BZR-Rns populations (χ^2^ = 9.74; df = 2; *p* = 0.007). The subsequent pairwise survival comparisons yielded no significant differences (Figure 1B).

### 3.3. Fertility Life Table

The REC-S and BZR-R populations exhibited a greater R_0_ compared to the progenies F1 and F1′. Conversely, the BZR-Rns exhibited a R_0_ equivalent to that of the parental and progeny populations (Table 3).

The REC-S population exhibited the lowest T, succeeded by the resistant population BZR-R, BZR-Rns populations and progeny F1 (Table 2). The progeny and BZR-Rns populations exhibited the lowest r_m_ compared to the BZR-R populations, while the REC-S population demonstrated the highest estimated r_m_. For REC-S, BZR-R, and progeny F1′, the DT was lower, whereas the progenies F1 and BZR-Rns required more time (Table 3).

The relative fitness calculation resulted that the BZR-R, the backcross progenies (F1 and F1′), and the BZR-Rns population were not as fit as the REC-S population (Rf < 1.0) (Table 3).

## 4. Discussion

Field resistance of DBM larvae to lufenuron was first reported in Brazil by our group [7] because of successive applications of this insecticide and other IGDs on Brassicaceae crops. The observed RR in the studied population (BZR-R) exceeded that of the susceptible population by a thousand times, indicating an increase in resistance compared to previous studies [7,8]. Data on insect resistance to IGDs, such as lufenuron, are of general interest because the mode of action (MOA 15) is related to a physiological process required by insect larvae to grow, and it is considered rare [33]. The findings presented here aim to compare the life-history parameters of the REC-S, BZR-R, and BZR-Rns populations over a period of time, along with their reciprocal crosses, to identify any fitness costs associated with the resistance.

The BZR-Rns population exhibited a slight decline in the LC_50_ relative to the BZR-R population, with a reduction of 1.8 times. The data indicate that the resistance of *P. xylostella* to lufenuron appears stable, and this corroborates with previous studies linking this resistance to mutations in the CHS1 gene (chitin synthase), particularly the I1042M mutation [3,5,8].

Previous studies on *P. xylostella* showed that populations that are resistant might express fitness costs. Resistant populations demonstrated this by exhibiting longer larval development times and lower body masses in the instars [20,21,22,23]. However, our results demonstrate that, in fact, there are no fitness costs in the resistant population, which maintains its fitness. On the other hand, the progeny resulting from crosses between resistant and susceptible individuals exhibited lower relative fitness and low levels of resistance. This finding suggests that, although the resistant population remains unaffected, the offspring may face potential compromises. When crossing DBM parents that are resistant (BZR-R) and susceptible (REC-S), heterozygotes may be relatively prevalent in the progenies. This observation supports the early phases of insecticide selection, where the fitness costs in heterozygote-resistant populations are more significant than those in homozygote-resistant populations [33,34]. Considering that site mutation is the primary cause of this DBM population’s resistance to lufenuron [8], constitutive low expression and target-site modification in heterozygotes can lead to fitness costs, particularly if the gene is essential to survival or if the change results in a near loss of gene function [33]. Furthermore, any genetic modification could have pleiotropic effects on other features, leading to a change in physiology and possibly behaviour that impairs the individual, affecting the natural population’s ability to survive and reproduce [33,34,35].

In this study, the larval period was longer in the resistant population compared to the susceptible one, especially in the progenies. Furthermore, the weight of pupae in the susceptible population was greater than that of pupae from the resistant population, as were the weights of the progenies obtained through crossing. Similar results for *P. xylostella* were observed when comparing the R_0_ of progenies derived from a susceptible and an abamectin-resistant population, with fitness values of 0.50 and 0.53, respectively [24]. Additionally, previous research has shown that the progeny can suffer transgenerational effects, such as increased developmental time and decreased fertility [20]. In this study, although the effect on the resistant population was not as pronounced, the results suggest the presence of transgenerational effects that may influence progeny fitness.

The fitness of progenies, particularly when compared to susceptible parents, helps us to understand the evolution of resistance in the field and, consequently, outlines strategies to mitigate resistance selection. Progenies frequently demonstrate resistance during the initial phases of evolution in the field [36]. This analysis is especially relevant given the recessive nature of resistance to lufenuron in *P. xylostella,* as demonstrated by the LC_50_ values obtained for both parents and progeny in this study. A prior study has also shown that this resistance is autosomal (i.e., not sex-linked) and incompletely recessive [8]. Furthermore, offspring of the DBM population exhibiting resistance to chlorantraniliprole experienced transgenerational consequences, including prolonged development time and decreased fertility [20]. The results suggest that the progenies’ resistance to lufenuron incurs an adaptation cost, leading to disadvantages relative to the susceptible parents, as shown by a fitness estimate of less than 1.0.

Regarding the resistant populations (BZR-R and BZR-Rns), fitness was higher (0.87 and 0.74, respectively), showing similar developmental rates. These data reinforce the relative fitness of *P. xylostella* that are resistant to lufenuron in terms of stability; however, they contrast with the fitness value shown by a population with a high level of resistance to tebufenozide, for which the value was only 0.3 [30].

These aspects must be considered when managing insecticide resistance. Effective implementation of insecticide rotation as a management strategy is crucial, as it reduces the fitness of progenies in the absence of selection pressure. This can help delay resistance evolution and, consequently, reduce the number of resistance alleles, extending the lifespan of the molecules used [37].

## 5. Conclusions

The study’s findings show that (i) *P. xylostella* is not negatively affected by being resistant to the insecticide lufenuron; (ii) this species’ resistance to lufenuron is stable and recessive; (iii) offspring from crosses between susceptible and resistant parental populations are less fit than their parents; (iv) strategies such as preserving refuge areas for susceptible insects are essential for resistance management and prolonging the efficacy of lufenuron. These findings highlight the importance of integrated insecticide resistance management.

## Figures and Tables

**Table 1:** Susceptibility of *Plutella xylostella* to lufenuron on treated collard greens leaves. N stands for the number of larvae observed; F1′ and F1 refer to the progeny populations from reciprocal crosses between females (♀) and males (♂) from susceptible (REC-S) and resistant (BZR-R) populations, and BZR-Rns stands for the resistant population without selection pressure for four successive generations.

Population	n	χ^2^ (df) ^1^	Slope ± SE ^2^	LC_50_ (FL 95%) ^3^	RR (FL95%) ^4^
REC-S	411	9.1 (11)	1.23 ± 0.07	0.71 (0.49–1.00)	1.00 (0.60–1.65)
BRZ-R	266	1.65 (5)	4.19 ± 0.93	870.5 (629.97–1040.15)	1224.26 (804.63–1862.73)
♀S × ♂R (F1′)	384	14.33 (10)	0.59 ± 0.07	2.12 (0.68–4.92)	2.98 (1.35–6.56)
♀R ×♂S (F1)	388	15.34 (10)	0.48 ± 0.06	3.80 (0.98–10.23)	5.35 (2.24–12.74)
BZR-Rns	210	3.59 (4)	2.19 ± 0.28	479.37 (362.51–629.25)	674.19 (430.20–1056.56)

^1^ Chi-square test and degree of freedom (*p* > 0.05). ^2^ Slope of the fitted concentration–mortality equation and standard error. ^3^ Lethal concentration 50% (mg lufenuron L^−1^) and respective 95% probability fiducial limits. ^4^ RR: ratio of the LC_50_ estimate between resistant and susceptible populations calculated using the lethal ratio test [26].

**Table 2:** Life history characteristics of *Plutella xylostella* from different populations and progenies (F1 and F1′) from reciprocal crosses between females (♀) and males (♂) from BZR-R, REC-S, and resistant population without selection pressure after four successive generations (BZR-Rns).

Characteristics	Populations and Progenies				
	REC-S	BZR-R	♀R × ♂S (F1)	♀S × ♂R (F1′)	BZR-Rns
Larval stage, days	7.9 ± 0.05 a	8.6 ± 0.09 b	9.5 ± 0.12 c	9.4 ± 0.09 c	9.2 ± 0.04 c
Pupal stage, days	3.7 ± 0.06 ab	3.9 ± 0.05 b	3.5 ± 0.09 a	3.6 ± 0.08 ab	3.6 ± 0.05 a
Weight of pupa, mg	6.2 ± 0.11 a	5.8 ± 0.10 ab	5.7 ± 0.19 b	5.8 ± 0.13 ab	5.8 ± 0.11 ab
♀ longevity, days	10.9 ± 0.93 a	13.2 ± 1.30 a	12.8 ±0.72 a	11.9 ± 1.44 a	10.5 ± 1.11 a
♂ longevity, days	14.3 ± 1.47 ab	19.4 ± 1.31 a	13.2 ± 1.28 b	10.1 ± 0.93 b	13.3 ± 1.13 b
No. of eggs per♀	241.3 ± 14.05 ab	213.8 ± 12.10 ab	265.8 ± 9.21 a	205.1 ± 17.42 b	188.4 ± 19.27 b
No. of larvae	153.1 ± 19.93 ab	145.6 ± 6.34 ab	175.2 ± 12.33 a	120.9 ± 13.89 ab	113.3 ±17.32 b

Means (±SE) followed by the same letter within each row do not differ among populations according to Tukey’s HS test (α = 0.05).

**Table 3:** Life table parameters and relative fitness of *Plutella xylostella* from different populations and progenies (F1 and F1′) from reciprocal crosses between females (♀) and males (♂) from BZR-R, REC-S, and resistant population without selection pressure after four successive generations (BZR-Rns).

Life Table Parameters	Populations and Progenies				
	REC-S	BZR-R	♀R × ♂S (F1)	♀S × ♂R (F1′)	BZR-Rns
R_0_ (♀*♀^−1^)	53.2a (46.2–60.1)	46.5a (40.6–52.4)	28.2b (26.0–30.4)	34.1b (27.6–40.5)	39.4ab (30.3–48.5)
T, days	16.4c (16.1–16.6)	17.5b (17.1–17.9)	18.4a (18.1–18.8)	17.8ab (17.4–18.3)	18.8ab (17.9–19.7)
r_m_ (♀/♀*day^−1^)	0.24a (0.23–0.25)	0.21b (0.21–0.22)	0.18c (0.17–0.18)	0.19c (0.18–0.20)	0.19c (0.18–0.20)
DT, days	2.86d (2.74–2.97)	3.16c (3.03–3.28)	3.83a (3.76–3.90)	3.50b (3.31–3.68)	3.54a (3.30–3.78)
Relative fitness ^1^	-----	0.87	0.52	0.64	0.74

Mean values followed by different letters within rows indicate significant differences among populations by the fiducial limit overlapping rule at 95% probability, calculated through Jackknife’s method [27]. ^1^ Relative fitness = R_0_ (of the population to be compared)/R_0_ (REC-S) [30].

## Data Availability

The analysis protocols used during the current study are available from the corresponding author on request, and the raw data used can be downloaded from https://data.mendeley.com/datasets/fdjnhpmdcm/1, accessed on 1 October 2024.
